# Raman enhancement by graphene-Ga_2_O_3_ 2D bilayer film

**DOI:** 10.1186/1556-276X-9-48

**Published:** 2014-01-28

**Authors:** Yun Zhu, Qing-Kai Yu, Gu-Qiao Ding, Xu-Guang Xu, Tian-Ru Wu, Qian Gong, Ning-Yi Yuan, Jian-Ning Ding, Shu-Min Wang, Xiao-Ming Xie, Mian-Heng Jiang

**Affiliations:** 1 State Key Laboratory of Functional Materials for Informatics, Shanghai Institute of Microsystem and Information Technology, Chinese Academy of Sciences, 865 Changning Road, Shanghai 200050, People's Republic of China; 2 Center for Low-Dimensional Materials, Micro-Nano Devices and System, Changzhou University, Changzhou 213164, China; 3 Ingram School of Engineering, Texas State University, San Marcos, TX 78666, USA; 4 Materials Science, Engineering and Commercialization Program, Texas State University, San Marcos, TX 78666, USA

**Keywords:** Graphene, Raman enhancement, Gallium oxide, Chemical vapor deposition

## Abstract

2D β-Ga_2_O_3_ flakes on a continuous 2D graphene film were prepared by a one-step chemical vapor deposition on liquid gallium surface. The composite was characterized by optical microscopy, scanning electron microscopy, Raman spectroscopy, energy dispersive spectroscopy, and X-ray photoelectron spectroscopy (XPS). The experimental results indicate that Ga_2_O_3_ flakes grew on the surface of graphene film during the cooling process. In particular, tenfold enhancement of graphene Raman scattering signal was detected on Ga_2_O_3_ flakes, and XPS indicates the C-O bonding between graphene and Ga_2_O_3_. The mechanism of Raman enhancement was discussed. The 2D Ga_2_O_3_-2D graphene structure may possess potential applications.

## Background

The assembly of graphene with other nanostructures can broaden the graphene applications. Considerable investigation has been carried out on the assembly of graphene powder with functional materials, such as reduced graphene oxide-TiO_2_ composites to enhance photocatalytic degradation activity [1-3], graphene-MoS_2_ for high effective hydrogen evolution reaction [4,5], and graphene-Co_3_O_4_/Fe_3_O_4_ as anode material for lithium ion battery [6-9]. Two typical approaches for the assembly are extensively used. One is the hydrothermal approach wherein graphene oxide powder and other precursors are mixed with water or organic solvents and then undergo a hydrothermal process [1-5,7]. The other approach is the mixing of reduced graphene oxide with the other materials followed by post-thermal reduction [6,8,9]. In addition, the assembly of functional materials on continuous graphene films synthesized by chemical vapor deposition (CVD) has been attracting attention gradually, owing to the high quality of graphene films. For example, a thin amorphous aluminum oxide layer was deposited on a graphene film through atomic layer deposition to selectively decorate and passivate the edges of graphene nanoribbons [10]. ZnO was also deposited on CVD graphene, and the composite could be applied to a solar cell to replace ITO [11]. A graphene/single-wall carbon nanotube hybrid was synthesized by a facile catalytic CVD growth on layered double hydroxide at high temperature, and the hybrid structure exhibited excellent performance in Li-S batteries with a high capacity [12].

Ga_2_O_3_ is a deep ultraviolet transparent semiconductor [13,14], which has several different crystalline phases, including α-, β-, γ-, δ-, and ϵ-Ga_2_O_3_[15]. Among these phases, monoclinic structured β-Ga_2_O_3_ is the most stable form with a wide bandgap of 4.9 eV [14]. Because of its good luminescence properties, β-Ga_2_O_3_ has a useful application in phosphors. The hybrid structure of graphene and Ga_2_O_3_ is promising for flexible display devices by exploiting high conductivity and flexibility of graphene and the good luminescence of Ga_2_O_3_. Herein, we report a simple and one-step CVD process to assemble β-Ga_2_O_3_ flakes on a continuous graphene film. The morphology of the composite was characterized by optical microscopy (OM), field emission scanning electron microscopy (FESEM), Raman spectroscopy, and energy dispersive spectroscopy (EDS) mapping. The assembly mechanism was discussed. Importantly, it was found that the as-grown β-Ga_2_O_3_ flakes enhanced the intensity of the graphene Raman signal ten times. The possible Raman enhancement mechanism is proposed.

## Methods

A 0.2 g Ga with 7 N purity from UMC was laid on a designed quartz bowl, loaded into the quartz tube, and heated to 1,000°C under the protection of 200 sccm Ar and 2 sccm H_2_. The sample was annealed at 1,000°C for 1 h to remove the surface oxide. The graphene film was synthesized through CVD for only 3 min under 200 sccm Ar flow with 1.5 sccm CH_4_. After the growth of the graphene, the carbon source was turned off and the temperature was kept at 1,000°C for 30 min. Then, the furnace cover was opened for fast cooling down to room temperature either immediately at 1,000°C or after controllably cooling (approximately 10°C/min) down first to 800°C, 600°C, and 400°C, respectively. The samples were placed in a refrigerator for several hours for solidification before characterization since the melting temperature of gallium is about 29.8°C and has strong supercooling effects, causing its liquid state at room temperature.

OM (Leica Microscopy DM6000M, Germany) was used for the preliminary exploration. Raman microprobe spectroscopy (Thermo Fisher DXR, Waltham, MA, USA) with an Ar^+^ laser (excitation wavelength 532 nm, 1 to 5 mW, and beam spot approximately 1 μm), FESEM (FEI NOVA NanoSEM with an operating voltage of 5 kV, Hillsboro, OR, USA), and energy dispersive spectroscopy (EDS) analysis (Oxford X-max 80, Oxfordshire, UK) were employed to characterize the samples. X-ray photoelectron spectroscopy (XPS, Thermo Scientific ESCALAB 250) with a monochromatized Al Kα X-ray source (1,486.6 eV photons) was used to study the bonding between graphene and Ga_2_O_3_. A Shirley background was removed from the atomic spectra prior to deconvolution. We tried to conduct an atomic force microscopy and transmission electrical microscopy in order to directly characterize the thickness and the interface between the layers, but the graphene film decorated by Ga_2_O_3_ flakes curled up after removing the Ga substrate, rendering high-quality sample impossible.

## Results and discussion

**Figure 1:**
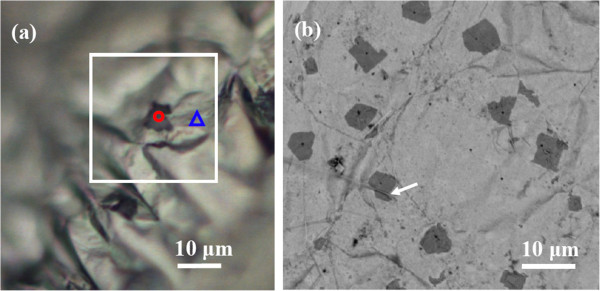
**OM (a) and FESEM (b) images of Ga**_**2**_**O**_**3 **_**sheets on the graphene surface.**

The CVD graphene growth on liquids, including Ga, Sn, and In, has been reported in our previous work [16]. Liquid Ga is very effective for graphene formation, and it can remain liquid under room temperature. The solid ultrathin graphene film on liquid Ga surface under room temperature is very unique. However, during the CVD process, Ga can react with oxygen to form oxide due to its high reactivity. It is found that Ga_2_O_3_ flakes could grow on graphene films by controlling the cooling step after graphene-film growth. When the tube furnace cover was opened immediately at 1,000°C for fast cooling after cutting off CH_4_ gas and keeping Ar flow, no Ga_2_O_3_ flakes were observed on the sample. In contrast, the Ga_2_O_3_ flakes could be observed by OM on the samples, which were cooled down to 800°C with a rate of approximately 10°C/min in Ar and then fastly cooled down to room temperature by opening the furnace cover, as shown in Figure 1a. The oxygen may be released by quartz or the residual oxygen in the CVD quartz tube. The Ga surface is covered by a continuous graphene film with several dark polygons under OM and FESEM. The as-prepared sample was a millimeter-sized liquid drop, and after freezing, wrinkles appeared on the convex surface, causing defocus somewhere under OM [16]. To further confirm the existence and distribution of irregular polygons, FESEM was conducted, as shown in Figure 1b. According to both OM and FESEM measurements, the lateral size of the polygons is around 1 to 10 μm. It is hard to determine the thickness of these polygon flakes. However, these flakes should be very thin and flexible since the flakes adhere well to graphene and conformally cover the graphene wrinkles, as the arrow indicates in Figure 1b.

**Figure 2:**
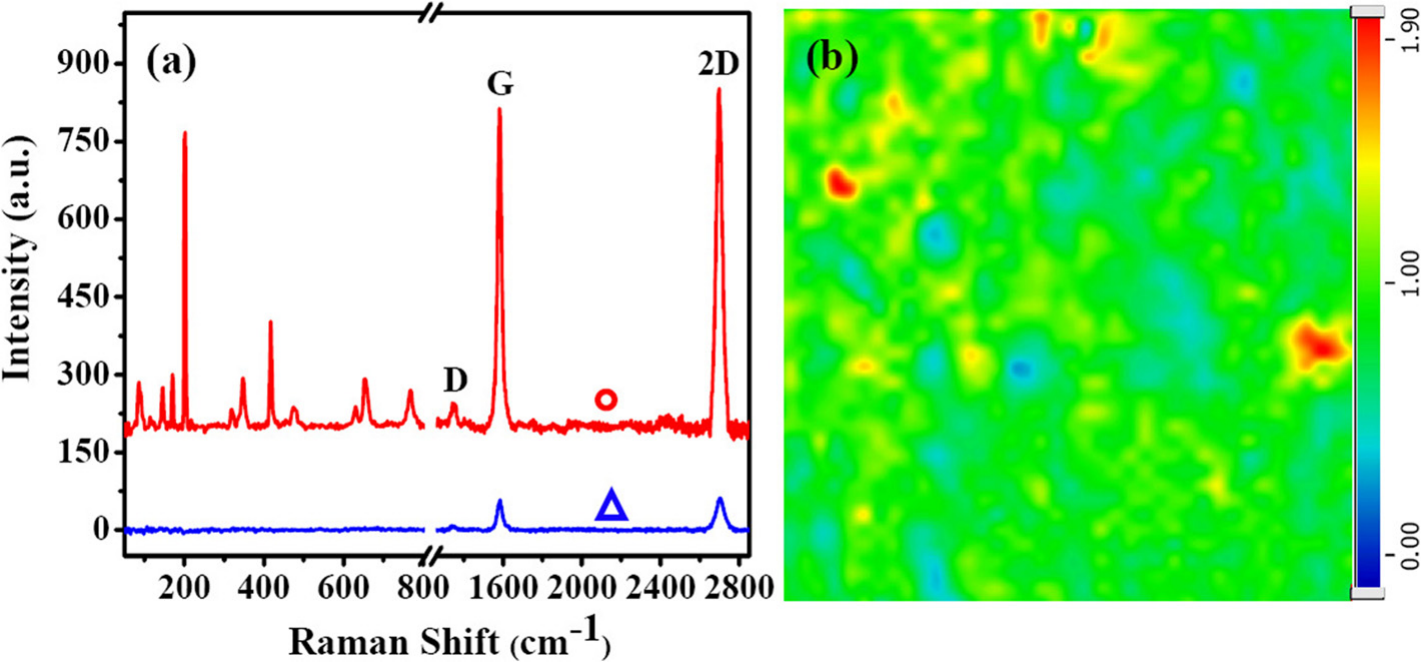
**Raman spectra and Raman mapping. (a)** Typical Raman spectra for the as-grown graphene film at the locations with and without Ga_2_O_3_ flakes. **(b)** The Raman mapping of *I*_2D_/*I*_G_ on the area marked as a white square in Figure 1a.

Raman measurements confirmed the formation of continuous graphene film and Ga_2_O_3_ flakes. Figure 2a shows the Raman spectra on the locations marked by a blue triangle and a red circle in Figure 1a. The blue spectrum from the continuous film shows the typical Raman features of graphene with 2D and G peaks. The defect-related D peak is very weak. The red spectrum, measured on the polygon flakes, also shows the typical Raman features of graphene. However, in the Raman shift range of 60 to 800 cm^−1^, more than ten additional peaks appear. These characteristic peaks correspond to β-Ga_2_O_3_[14,17]. The enlarged Raman spectra of graphene and Ga_2_O_3_, as well as the comparison of their peak positions with bulk Ga_2_O_3_ powders, are presented in Additional file 1: Figures S1 and S2 and Table S1. These Raman results confirm that the continuous film is graphene and that the dark polygon is β-Ga_2_O_3_ flakes. A Raman mapping on the area marked as a white square in Figure 1a is shown in Figure 2b. The homogenous color distribution indicates that β-Ga_2_O_3_ flakes do not change the ratio of *I*_2D_/*I*_G_ of graphene, i.e., the formation of the β-Ga_2_O_3_ sheets seems to have no effect on the continuity and thickness of graphene.

**Figure 3:**
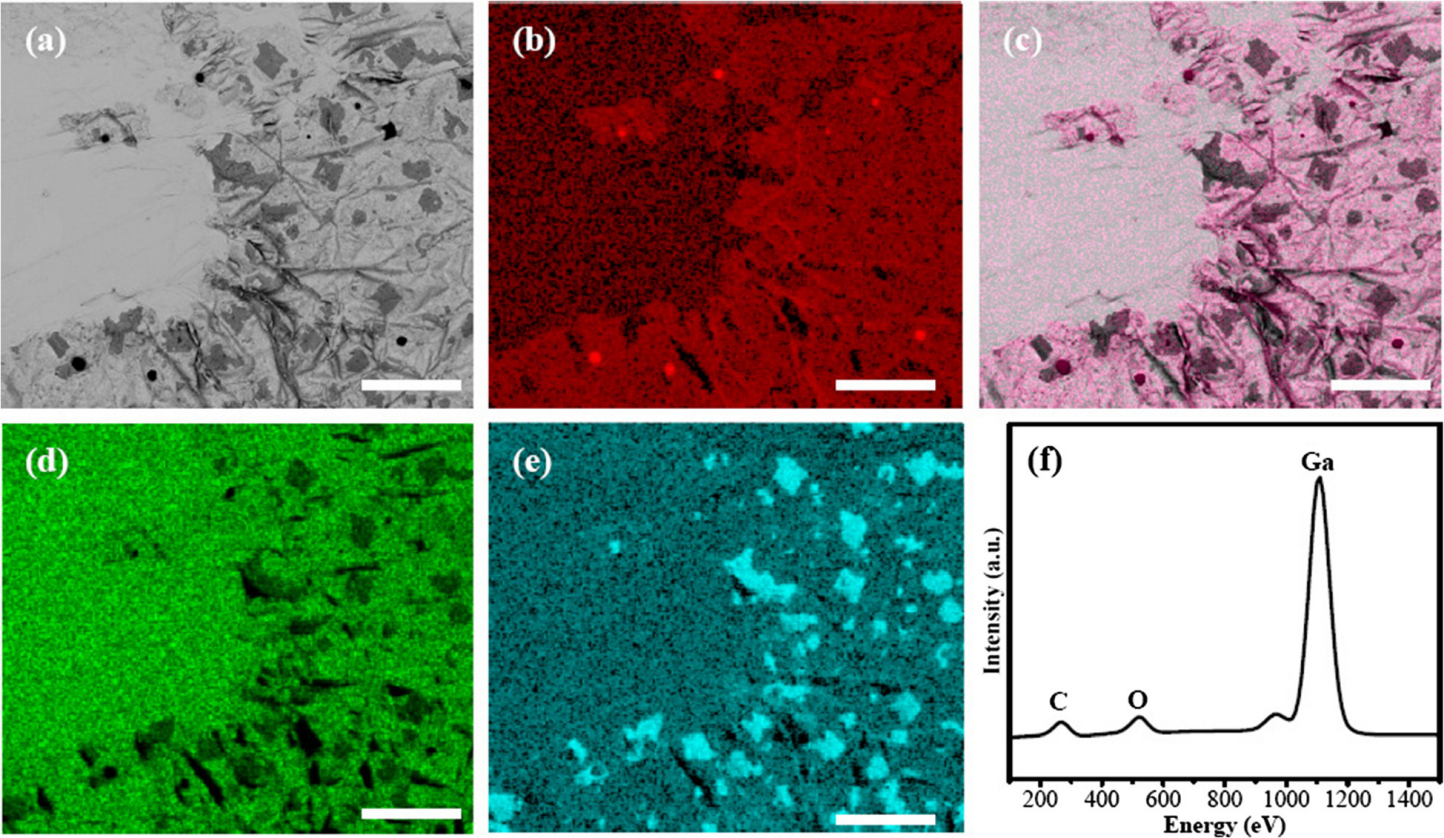
**FESEM images and EDS mapping and analysis. (a)** FESEM image of 2D Ga_2_O_3_-2D graphene composite, **(b)** the EDS mapping of element C, **(c)** combination of morphology and element C distribution, **(d)** and **(e)** EDS mappings of elements Ga and O, respectively, and **(f)** EDS element analysis. The scale bar is 50 μm.

During the FESEM characterization, EDS mapping was employed to further confirm the components and element distribution. Figure 3a shows the surface morphology of a sample with an area of approximately 205 × 240 μm^2^. The flat and clear area at the left is the bare Ga surface, which is exposed due to volume expansion during the solidification of liquid Ga and/or due to the different thermal expansion coefficients between Ga and graphene [16]. The EDS mapping of carbon on the same area in Figure 3b shows good match with the morphology of the graphene film, as shown in Figure 3c. The EDS mapping of carbon, together with following Raman and XPS measurements, confirms a graphene film on the Ga surface. At the locations of the β-Ga_2_O_3_ flakes, the carbon signal does not decrease. This result indicates that the β-Ga_2_O_3_ flakes do not hinder the formation of the continuous graphene film and is consistent with the above Raman analysis. The red dots in Figure 3b correspond well to the dark dots in Figure 3a, and these carbon dots may be caused by amorphous carbon accumulation during the CVD process. The element mapping of Ga and O in Figure 3d,e directly confirms the formation of the Ga_2_O_3_ flakes, since the shape and position of Ga and O distribution is consistent with the polygons in Figure 3a. The statistical element analysis in Figure 3f also supports the element mapping results.

**Figure 4:**
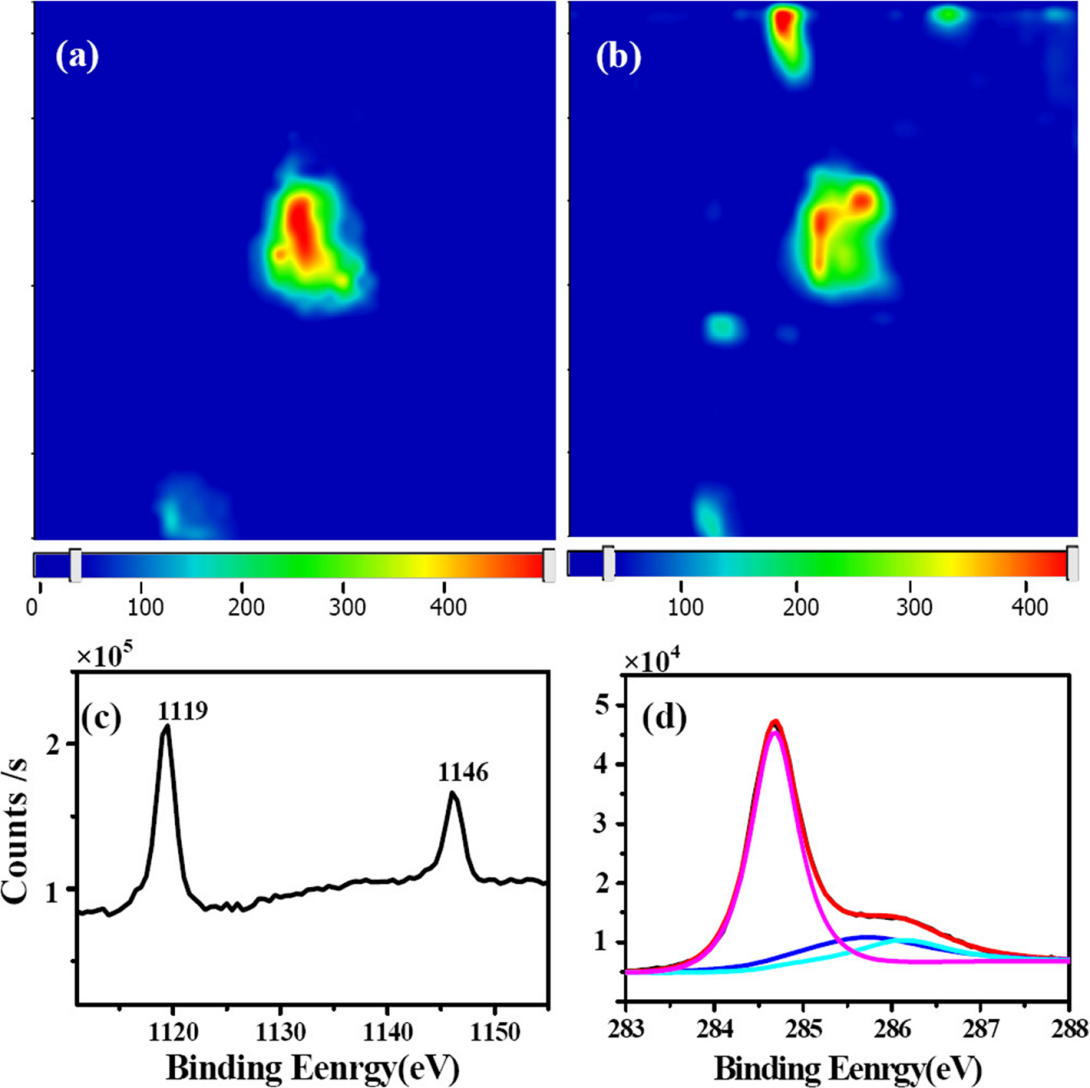
**Raman mapping and XPS spectra.** Raman mapping of **(a)** 1,583-cm^−1^ peak and **(b)** 202-cm^−1^ peak. **(c)** XPS spectra of Ga2p and **(d)** XPS spectra of C1s. The black curve is the original data, and the red is the fitting curve. The pink, dark blue, and sky blue curves are the fitting peaks on 284.7, 285.7, and 286.2 eV, respectively.

The above results from OM, FESEM, Raman, and EDS mapping confirm the formation of a special β-Ga_2_O_3_-graphene composite on Ga. This structure may have potential applications due to the 2D-2D assembly. More importantly, we found the interesting property of the graphene Raman enhancement by the β-Ga_2_O_3_ flakes, as shown in Figure 2a and Additional file 1: Figure S1. The intensity of G and 2D peaks increases to more than ten times, although the *I*_G_/*I*_2D_ ratio does not change. Figure 4a shows the G peak mapping image of a graphene area marked by the white box in Figure 1a, and the 2D peak mapping has the similar image. The positions of the graphene Raman enhancement correspond with the distribution of Ga_2_O_3_. Through comparing two images of Figure 4a,b, it was found that not all the Ga_2_O_3_ flakes have same efficiency to enhance the graphene Raman signal. This phenomenon indicates that the graphene Raman enhancement may be related to the thickness of Ga_2_O_3_.

The surface-enhanced Raman scattering (SERS) has been extensively investigated [18-20]. The charge transfer between the two contacted materials is the chemical mechanism of Raman enhancement [21,22]. For the graphene Raman enhancement by β-Ga_2_O_3_ flakes, the charge transfer is confirmed by the Raman and XPS data. In the Raman spectra of Figure 2a and Additional file 1: Figure S1, the G-band has a downshift of approximately 2 cm^−1^ (1,584 to 1,582 cm^−1^) at the locations of the β-Ga_2_O_3_ sheets, which indicates the charge transfer between the graphene and β-Ga_2_O_3_, and β-Ga_2_O_3_ as an electron donor to the graphene [23]. In addition, the work function of graphene (4.2 eV) [24] and Ga_2_O_3_ (4.11 ± 0.05 eV) [25] is proximity; this is consistent with the slight downshift of the graphene G-band. In addition to the Raman data, the XPS data also support the CM mechanism. Additional file 1: Figure S3 shows the XPS spectrum, showing a general scan in the energy range from 0 to 1,200 eV. The peaks of the core levels of Ga2p, Ga3s, Ga3p, Ga3d, and Ga LMM peaks, as well as the O1s, OKLL, and C1s, were detected. Additional file 1: Figure S4 shows the O1s peak with a binding energy around 532 eV, which is corresponds to the Ga-O bonding of Ga_2_O_3_. The two peaks of Ga2p for the Ga-O bondings are also clearly observed in Figure 4c [26,27]. A high-resolution XPS C1s spectrum is given in Figure 4d. Using a suitable application of Gaussian and Lorentzian functions, the C1s peak can be decomposed into three apparent spectral components at 284.7, 285.7, and 286.2 eV. The main peak at 284.7 eV corresponds to the graphite-like *sp*^2^ C, and the 285.7 and 286.2 eV peaks are attributed to *sp*^3^ carbon and C-O bonds [24,28]. The XPS data is consistent with the aforementioned Raman and EDS results to confirm the Ga_2_O_3_-graphene structure, and XPS also presents the evidence of the C-O bands, which confirms the negative-charge doping effect from the Ga_2_O_3_ sheets on the graphene film. Due to chemical doping, polarizability of graphene is increased, leading to an increase in the Raman scattering cross-section [29].

It is necessary to discuss the formation mechanism of Ga_2_O_3_-graphene. Ga itself is very reactive and can react with most materials under high temperature. In the periodic table of elements, Ga and Al are in the same main group and have similar characteristics. Analogously, Ga can form a continuous and compact oxidized layer in air, which impedes further oxidation of Ga. Therefore, we need to remove the very thin surface oxide before graphene growth through pre-annealing in Ar/H_2_ atmosphere. During the CVD graphene growth, hydrogen is hazardous for the graphene formation [16], and H_2_ was not applied during the growth stage. We proposed that the graphene grows on the surface of the liquid Ga at first and then the Ga_2_O_3_ sheets come into being on the graphene during the cooling process, as shown in the schematic illustration of Additional file 1: Figure S4. The O element comes from the oxygen residue in the tube, and the C-O bonds which have been evidenced by XPS are the defects on the 2D graphene film. These defects play an important role for the growth of Ga_2_O_3_ on graphene because they will act as nucleation points of Ga_2_O_3_ since Ga atoms in the vapor will obviously prefer O as a bonding target, not the carbon atoms.

This mechanism is supported by two evidences. The first one is that after immersing the samples of 2D Ga_2_O_3_-2D graphene into dilute hydrochloric acid for 1 h at room temperature, the Ga_2_O_3_ sheets will disappear. If the graphene covers and protects the Ga_2_O_3_ sheets, it is hard to remove Ga_2_O_3_ in hydrochloric acid. Another evidence is related to the cooling process. We chose different rapid cooling starting points of 800°C, 600°C, and 400°C. More polygon sheets or granules deposited on the graphene surface when the sample underwent longer cooling durations. According to the illustration depicted in Additional file 1: Figure S4, it is possible to control the deposition of Ga_2_O_3_ sheets on the graphene surface to form the special 2D Ga_2_O_3_ nanosheet-2D graphene sheet structure through a one-step CVD process. Compared to the general Raman enhancement by metals, such as silver and gold, the Ga_2_O_3_ nanosheets have remarkable thermal stability. Conversely, silver will oxidize excessively and becomes quenched within 36 h in the air [30]. The 2D graphene-Ga_2_O_3_ film can be transferred onto other targets and may be used as bio-substrate through SERS. The stability of the Ga_2_O_3_ nanosheets and the structure stability need to be further investigated.

## Conclusions

In summary, separated 2D thin Ga_2_O_3_ nanosheets, with a lateral size of 1 to 10 μm, on continuous 2D graphene film were synthesized by a one-step CVD process on liquid gallium substrate. The Raman and EDS mapping confirm the formation of the β-Ga_2_O_3_ sheets on the graphene surface. The formation mechanism was proposed as a β-Ga_2_O_3_ sheet formation after graphene synthesis during the cooling process. The graphene Raman enhancement over ten times was detected on the β-Ga_2_O_3_ sheets due to the charge transfer. The 2D-2D structure may have potential application in optical and electronic devices.

## Competing interests

The authors declare that they have no competing interests.

## Authors' contributions

YZ, X-GX, and G-QD carried on the experimental parts. Q-KY, G-QD, T-RW, and QG analyzed and interpreted the data. Q-KY and G-QD wrote the manuscript. N-YY, J-ND, S-MW, X-MX, and M-HJ were involved in the discussions and revision of the manuscript. All authors read and approved the final manuscript.

## Supplementary Material

Additional file 1 Raman data, XPS analysis, and proposed growth mode. Click here for file
