# Supplementary material for: Raman enhancement by graphene-Ga2O3 2D bilayer film
Source: Nanoscale Res Lett. 2014 Jan 28;9(1):48. doi: 10.1186/1556-276X-9-48 (PMC3906882; doi:10.1186/1556-276X-9-48)
Supplement: Additional file 1 — Raman data, XPS analysis, and proposed growth mode. [file 1556-276X-9-48-S1.doc]

**Raman Enhancement by Graphene-Ga_2_O_3_ 2D Bilayer Film**

Y. Zhu^1,2^, Q. K. Yu^3^, G. Q. Ding1[[1]](#footnote-2)*, X. G. Xu^1^, T. R. Wu^1^, Q. Gong^1^, N. Y. Yuan^2^, J. N. Ding^2^, S. M. Wang^1^, X. M. Xie^1^, and M. H. Jiang^1^

^1^State Key Laboratory of Functional Materials for Informatics, Shanghai Institute of Microsystem and Information Technology, Chinese Academy of Sciences, 865 Changning Road, Shanghai 200050, People’s Republic of China

^2^Center for Low-dimensional Materials, Micro-nano Devices and System, Changzhou University, Changzhou 213164, China

^3^Ingram School of Engineering, and Materials Science, Engineering and Commercialization Program, Texas State University, San Marcos, Texas 78666, United States

**Raman data**

Figure S1. Enlarged Raman peaks of G and 2D of graphene.

The graphene G-band downshifts from 1584.7 cm^-1^ to 1582.7 cm^-1^ when β-Ga_2_O_3_ sheet on the surface of graphene. At the same time, the 2D peak also has a downshift of 4 cm^-1^, 2 times of the G peak shift.

Figure S2. Enlarged Raman peaks of β-G_2_O_3_.

The Raman peaks range in 100-800 cm^-1^ can be assigned toβ-G_2_O_3_.Compared to the Raman peaks of G_2_O_3_ nanowires, the narrow full width at half maximum indicates the good crystalline fabricated G_2_O_3_ sheets on graphene.

Table 1 shows the Raman peak positions from present work and the Ref. 14 and 17. It is clear that our samples can be well assigned to β-G_2_O_3_ since all the peaks can find its counterparts from bulk samples. It should be noted that 1-4 cm^-1^ upshift takes place for most peaks. This upshift may be induced by two effects: 1) the stain induced Raman shift. Ref. 17 has reported the Raman shift due to strains in the nanowires. Since Ga will expand during the cooling while β-G_2_O_3_ will contract, the β-G_2_O_3_ sheet may undergo a compression stress at room temperature. 2) It has been confirmed by XPS that the O inβ-G_2_O_3_ has chemical bonding with C in grpahene, which affects the vibration of Ga-O inβ-G_2_O_3_.

**Table S1. The peak positions of G_2_O_3_ sheets and bulk G_2_O_3_ powders from Ref. 14 and 17.**

| Bulk [14] | 142 | 167 | 198 | 320 | 344 | 415 | 473 | 627 | 651 | 765 |
| --- | --- | --- | --- | --- | --- | --- | --- | --- | --- | --- |
| Bulk [17] | 144 | 169 | 200 | 317 | 344 | 416 | 472 | 629 | 654 | 767 |
| This work | 146 | 171 | 202 | 319 | 348 | 417 | 474 | 630 | 653 | 769 |

**XPS analysis**

**(a)**

Figure S3. the XPS survey spectrum of Ga_2_O_3_-graphene structure.

The XPS spectra, scan range from 0 to 1200 eV, shows the peaks of the core levels of Ga2p, Ga3s, Ga3p, Ga3d, Ga LMM peaks, as well as the O1s, OKLL and C1s. These peaks confirm the formation of graphene and Ga_2_O_3_.

**The growth mode**

**Graphene**

**(a)**

**Ga**

**Quartz**

**Graphene**

**(b)**

**Ga_2_O_3_**

**Ga**

**Quartz**

Figure S4. Schematic illustration of feasible growth mechanism (a) continuous graphene film covers the liquid Ga surface (d) separated Ga_2_O_3_ sheets deposited on graphene during the sample cooling process.

1. ^^Author to whom correspondence should be addressed. Electronic mail: gqding@mail.sim.ac.cn. [↑](#footnote-ref-2)
